# The ideal approach for treatment of cT1N+ and cT2Nany esophageal cancer.: a NCDB analysis

**DOI:** 10.1186/s12885-021-08896-0

**Published:** 2021-12-15

**Authors:** Binhao Huang, Ernest G. Chan, Arjun Pennathur, James D. Luketich, Jie Zhang

**Affiliations:** 1 Department of Thoracic Surgery, Shanghai Chest Hospital, Shanghai Jiaotong University, Shanghai, China; 2 Department of Gastric Surgery, Fudan University Shanghai Cancer Center, Shanghai, China; 3 Department of Oncology, Shanghai Medical College, Fudan University, Shanghai, China; 4 Department of Cardiothoracic Surgery, University of Pittsburgh Medical Center, 200 Lothrop St, Suite C800, Pittsburgh, PA 15213 USA

**Keywords:** Esophageal cancer, Neoadjuvant therapy, Adjuvant therapy, National Cancer Database Analysis of Esophageal Cancer

## Abstract

**Background:**

Neoadjuvant therapy followed by surgery is recommended for locally advanced esophageal cancer. With the inaccuracies of clinical staging particularly for cT1N+ and cT2Nany tumors, some have proposed consideration of surgery followed by adjuvant treatment. Our objective is to evaluate the efficacy of neoadjuvant therapy vs surgery followed by adjuvant therapy, and to identify the ideal sequence of treatment in patients with cT1N+ and cT2Nany tumors.

**Methods:**

We performed an analysis utilizing the National Cancer Database (2006-2015) identifying all patients with cT1N+ and cT2Nany esophageal cancer undergoing esophagectomy. The treatment was stratified as: neoadjuvant therapy (NT), adjuvant therapy (AT) and combination therapy of neoadjuvant and adjuvant (CT) groups and outcomes were analyzed.

**Results:**

We identified 2795 patients with 81.9% (*n*=2289) receiving NT, 10.2% (*n*=285) AT, and 7.9% (*n*=221) CT. There were no significant differences noted in survival among AT, NT, and CT group in cT1N+(*P*=0.376), cT2N-(*P*=0.436), cT2N+(*P*=0.261) esophageal cancer by multivariate analysis using Cox regression model. This relationship held true in both squamous cell carcinoma and adenocarcinoma.

**Conclusion:**

In clinical T1N+, T2Nany patients, there was no evident superiority of NT over AT. Surgery followed by adjuvant therapy can be considered to be an alternative option in these patients. Further prospective studies are needed to validate these findings.

**Supplementary Information:**

The online version contains supplementary material available at 10.1186/s12885-021-08896-0.

## Background

Esophageal cancer remains the sixth leading cause of cancer-related mortality and the eighth most common cancer in the world with an incidence of over 450,000 people worldwide [1–5]. Including all stages, the overall 5-year survival esophageal cancer ranges from 15% to 25%, and the best outcomes are associated with disease diagnosed in the early stages [6]. Outcomes in patients with esophageal cancer are related to the propensity for metastases, even when tumours are superficial [5, 6]. Therefore, there is an urgent need to devise optimal treatment strategies for a multimodal approach.

In the Intergroup 0113 study, there were no differences noted in survival between patients who received chemotherapy followed by surgery vs surgery alone [7]. However, an important finding of Intergroup 0113 was the association between improved survival and those who had a superior response to chemotherapy. This was further corroborated by Pennathur et al in a phase II trial, where we used neoadjuvant chemotherapy, followed by surgery and additional adjuvant chemotherapy. This study revealed a significant improvement in median survival in patients who were downstaged as a result of the treatment (63.4 vs 21.5 months) [8]. In contrast, the Medical Research Council reported a significant improvement in survival in the chemotherapy arm in a randomized North American trial (OE02) [9]. The CROSS Trial which incorporated neoadjuvant chemoradiation strategy has shown a significantly improved survival and has been adopted widely [10].

While a neoadjuvant strategy has been widely used for locally advanced cancer, there has been concern about toxicity of treatment and its role in the treatment of earlier stage cancers. Unfortunately, there are a plethora of individual phase III studies have continued to show conflicting results, and there is controversy as to the optimal multimodality approach for esophageal cancer [3, 5, 7, 9, 11]. With the inaccuracies of clinical staging particularly for cT1N+ and cT2 tumors, some have proposed surgery followed by adjuvant treatment [12, 13]. Adjuvant therapy has its advantages in that definite surgery can be performed as upfront management, and subsequent treatment could be decided based on more reliable pathological information.

The objective of this study is to evaluate the efficacy of neoadjuvant therapy followed by surgery vs surgery followed by adjuvant therapy for cT1N+ and cT2Nany esophageal cancer.

## Methods

We initially performed a retrospective analysis utilizing the National Cancer Database (NCDB) to identify all patients with esophageal cancer, who underwent esophagectomy and received neoadjuvant, adjuvant, or a combination of both with surgery. We then focus in patients between the ages of 18 and 90 with cT1N+ and cT2Nany esophageal cancer. We included patients treated between the years of 2006 and 2015, as the sequence of additional therapy and surgery was not recorded prior to 2006. Supplemental Figure 1 shows a flowchart that summarizes inclusion and exclusion criteria. We included all patients diagnosed as cT1N+ and cT2Nany esophageal squamous cell carcinoma (ESCC) or adenocarcinoma (EAC). Patients were divided into three cohorts based on the sequence of their multimodal therapy regimen: neoadjuvant therapy (NT), adjuvant therapy (AT) and the combination therapy of neoadjuvant and adjuvant (CT).

### Variables

Included in the NCDB is a comprehensive social background information of patients, including insurance, facility, age, gender, race, Charlson-Deyo score, year of diagnosis, tumor location, histological subtype and clinical stages. Short-term outcomes were evaluated according to postoperative length of stay (LOS) and 30-day unplanned readmission, which may be a surrogate for postoperative complications. Overall survival (OS) was compared between three groups and subgroups as a long-term prognosis. Histological subtype and location were based on histology code of ICD-O3.

### Statistical analysis

Chi-square test was used for categorical variables and analysis of variance (ANOVA) for continuous variables. Kaplan-Meier curve and log-rank test was used to evaluate unadjusted OS between cohorts. Multivariable Cox regression model was built to obtain adjusted hazard ratio in different subgroups. All statistical analyses were performed in SPSS version 22.0 and figures were generated by Prism version 6.0. A *p* value of <0.05 was considered statistically significant for all analyses.

## Results

### Demographic and clinical characteristics

**Fig. 1:**
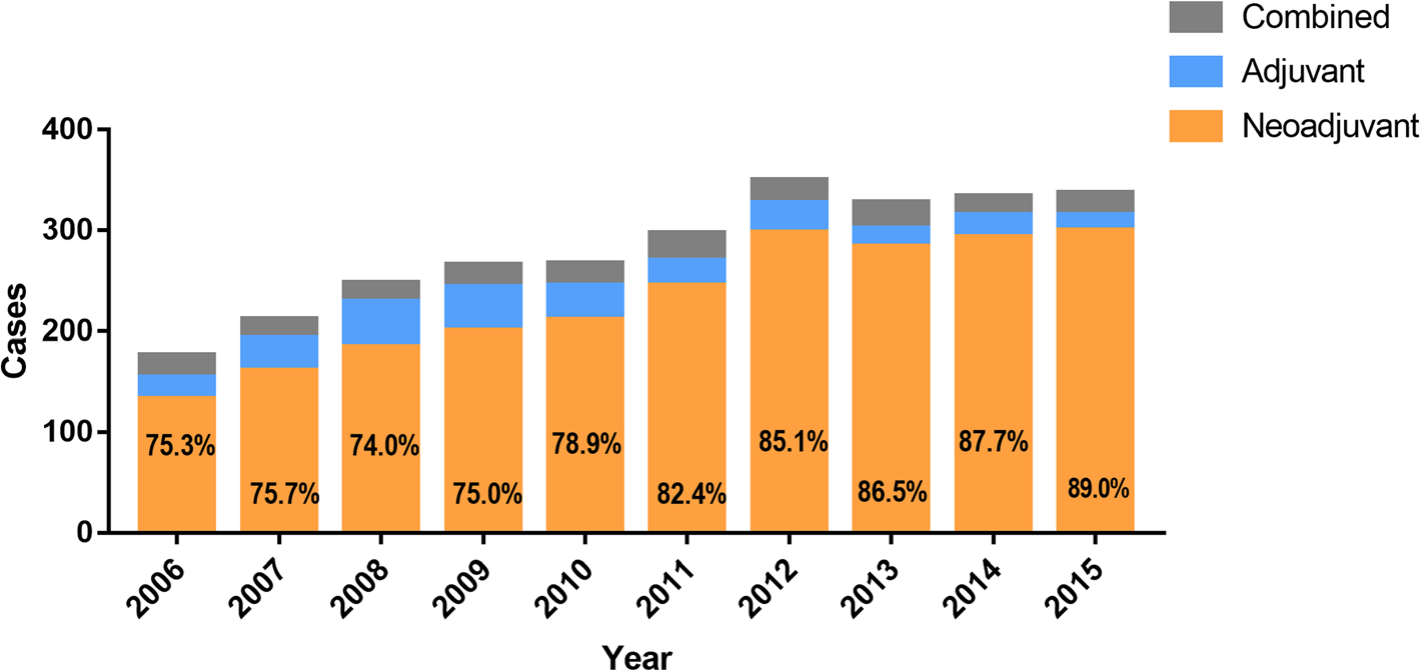
Trend of application of the different treatment strategies in 2006-2015 in the US

A total of 2795 patients were identified to fulfill the study inclusion/exclusion criteria (EAC in 2322 patients and ESCC 473 patients). When stratifying the patient population based on their multimodal therapy, 81.9% (*n*=2289) were treated with NT, 10.2% (*n*=285) with AT and 7.9% (*n*=221) with CT. Figure 1 displays the national trend of each group between 2006-2015 showing an increasing trend for neoadjuvant therapy.

### Analysis of postoperative complications stratified by approach

No significant differences were found between three groups regarding 30-day unplanned readmission (*p*=0.835) (Table 1). At the time of surgery, the rate of R0 resection was significantly lower in the AT group than in both NT and CT groups (84.6% vs 94.5% and 90.0%, *p*<0.001). Mean of LOS in NT, CT, and AT group were 13.4, 12.3, 12.0 days (*p*=0.248). 30- and 90- day mortality was significantly higher in the NT group when compared to AT and CT (30 -day mortality: 3.6% vs 0.7% vs 0.9%); 90 -day mortality: 8.1% vs 1.1% vs 4.1%, Table 2).

**Table 1:** Comparison of short-term outcome between 3 groups

	NT(***N***=2289)	AT(***N***=285)	CT(***N***=221)	*P value
**Negative surgical margin(R0)**	2162 (94.5%)	241 (84.6%)	199 (90.0%)	<0.001
**30-d unplanned readmission**	125 (5.5%)	18 (6.3%)	12(5.4%)	0.835
**Postoperative LOS, mean(range)**	13.4(0-125)	12.0(0-67)	12.3(0-69)	0.248

Short-term outcome as surgical margin and 30-d unplanned readmission was presented as N(percentage), Postoperative length of stay (LOS) was presented as mean (range)

*P value of surgical margin and readmission were derived from Chi-squared test. Comparison of postoperative LOS was conducted using analysis of variance (ANOVA)

**Table 2:** Comparison of 30 and 90 days mortality between 3 groups

	NT(***N***=2289)	AT(***N***=285)	CT(***N***=221)	***p*** value^**α**^	***p*** value^**β**^
**30-d mortality**	83 (3.6%)	2 (0.7%)	2 (0.9%)	0.004	1.000
**90-d mortality**	185 (8.1%)	3 (1.1%)	9 (4.1%)	<0.001	0.026

Data was presented as number and percentage for each group;

α:*P* value for comparison of NT, AT and CT;

β:*P* value for comparison of AT and CT

### Comparison of Neoadjuvant (NT) vs. Adjuvant (AT) vs. Combination therapy (CT) in specific subgroups

**Fig. 2:**
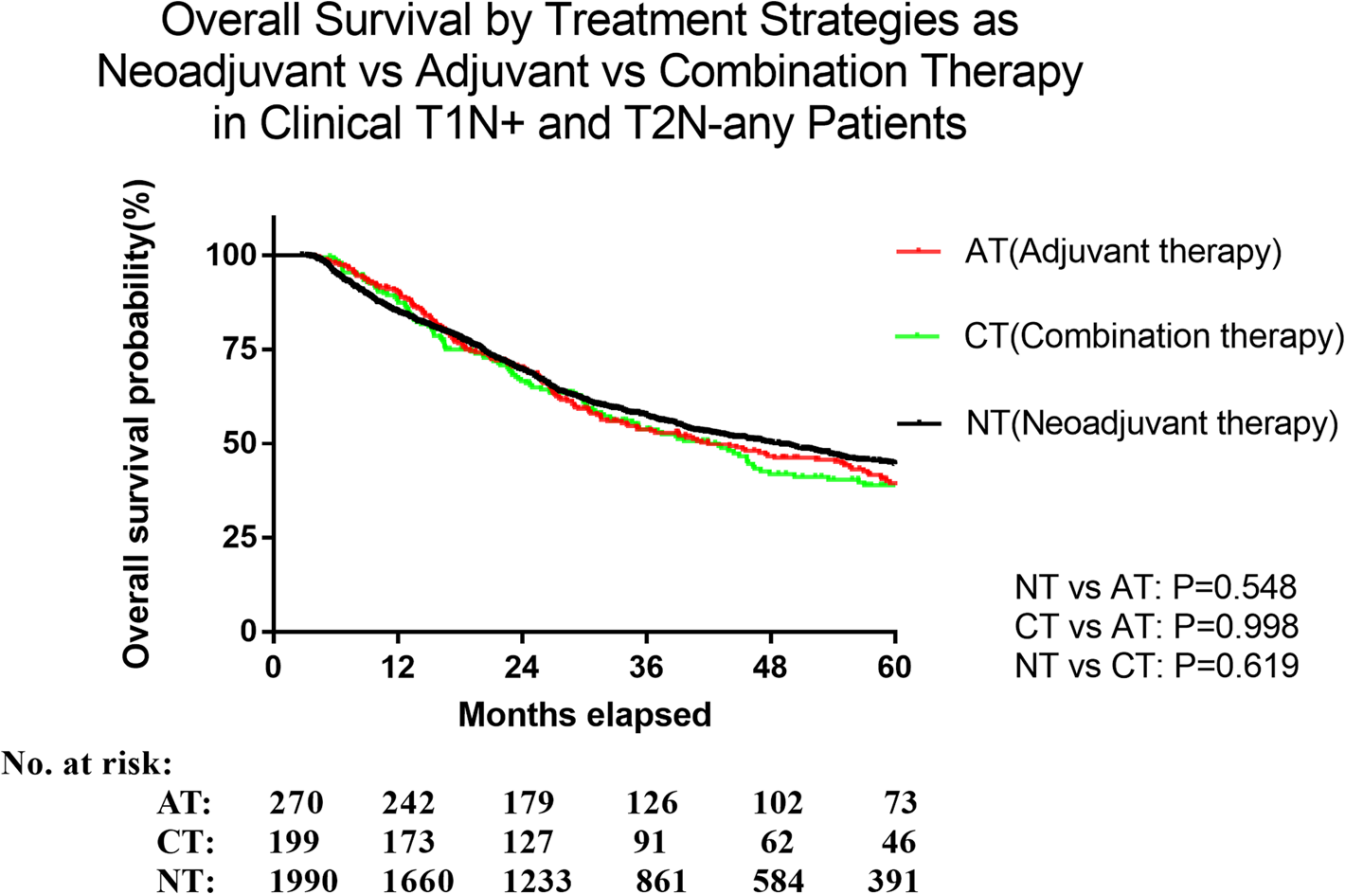
There was no difference in overall survival in patients who had clinical T1N+ and T2Nany between all three treatment groups. (NTvs AT, *P*=0.548, CT vs AT: *P*=0.998, NT vs CT *P*=0.619)

We compared the outcomes of NT vs. AT vs CT in specific subgroups and focused on any differences in the cT1N+ and cT2Nany group. There were no significant differences noted in OS between AT vs. NT groups in the cT1N+ and cT2Nany group (cT1N+(*P*=0.344), cT2N-(*P*=0.473), cT2N+(*P*=0.280)) by multivariate analysis using Cox regression model (Table 3, Fig. 2). The CT strategy did not show superiority over NT neither (cT1N+(*P*=0.332), cT2N-(*P*=0.205), cT2N+(*P*=0.997)). This relationship held true in both squamous cell carcinoma and adenocarcinoma groups (Supplemental 1). No differences in survival between AT and NT were noted in the subgroups except age above 76, Charlson-Deyo score =2. For some T2N0 patients, surgery alone strategy was also applied in clinical practice. In our study, survival outcome of surgery alone group was a little bit worse than AT, NT and CT group, but the difference did not reach statistical significance. (Supplemental 2).

**Table 3:** Subgroup analysis in multivariate Cox regression model for difference of overall survival in three cohorts

Subgroups	Strategy	Adjusted HR	95% CI of HR	Adjusted ***P*** value
**Reference**	NT	1.000	1.000	1.000
**Age**				
≤55	AT	1.074	0.712-1.619	0.735
	CT	1.077	0.734-1.581	0.704
56-65	AT	0.861	0.615-1.206	0.384
	CT	1.182	0.859-1.626	0.305
66-75	AT	1.039	0.740-1.459	0.825
	CT	0.771	0.476-1.250	0.292
>75	AT	2.399	1.304-4.413	0.005
	CT	3.723	0.966-14.344	0.056
**Sex**				
Male	AT	1.102	0.899-1.351	0.351
	CT	1.183	0.947-1.477	0.139
Female	AT	1.104	0.659-1.848	0.707
	CT	0.636	0.317-1.277	0.203
**Year of diagnosis**				
2006-2010	AT	1.053	0.839-1.321	0.657
	CT	1.027	0.775-1.360	0.855
2011-2015	AT	1.136	0.806-1.602	0.465
	CT	1.237	0.898-1.704	0.194
**Charleson score**				
0	AT	1.124	0.888-1.422	0.332
	CT	1.076	0.841-1.377	0.560
1	AT	0.902	0.623-1.306	0.586
	CT	1.305	0.835-2.039	0.242
≥2	AT	2.517	1.062-5.965	0.036
	CT	0.736	0.166-3.266	0.687
**Facility type**				
Community	AT	0.385	0.057-2.608	0.328
	CT	713.823	12.241-41626.965	0.002
Comprehensive Community	AT	1.078	0.759-1.531	0.675
	CT	1.140	0.805-1.615	0.460
Academic/Research	AT	1.079	0.842-1.383	0.547
	CT	1.256	0.943-1.674	0.120
Integrated Network	AT	1.127	0.529-2.401	0.757
	CT	0.787	0.302-2.047	0.623
**Histology type**				
ESCC	AT	1.542	0.988-2.407	0.057
	CT	0.805	0.453-1.431	0.459
EAC	AT	1.022	0.839-1.243	0.831
	CT	1.155	0.928-1.437	0.196
**c Stage**				
T1N+	AT	0.691	0.321-1.486	0.344
	CT	1.323	0.760-2.304	0.322
T2	AT	1.074	0.886-1.301	0.468
	CT	1.078	0.856-1.359	0.524
T2N0	AT	1.094	0.856-1.398	0.473
	CT	1.259	0.882-1.797	0.205
T2N+	AT	1.203	0.860-1.683	0.280
	CT	0.999	0.734-1.360	0.997
T1+T2	AT	1.018	0.846-1.225	0.849
	CT	1.122	0.909-1.385	0.283

Covariates in Cox model were all categorical variables, included age, sex, race, type of insurance, income, education, rurality, patient comorbidity, year of diagnosis, type of facility, histology type, tumor location and clinical T and N stage.

Adjusted *P* value and Hazard Ratio (HR) were derived from the model, putting NT group as reference

### Comparison of chemotherapy, radiotherapy and chemoradiotherapy

**Fig. 3:**
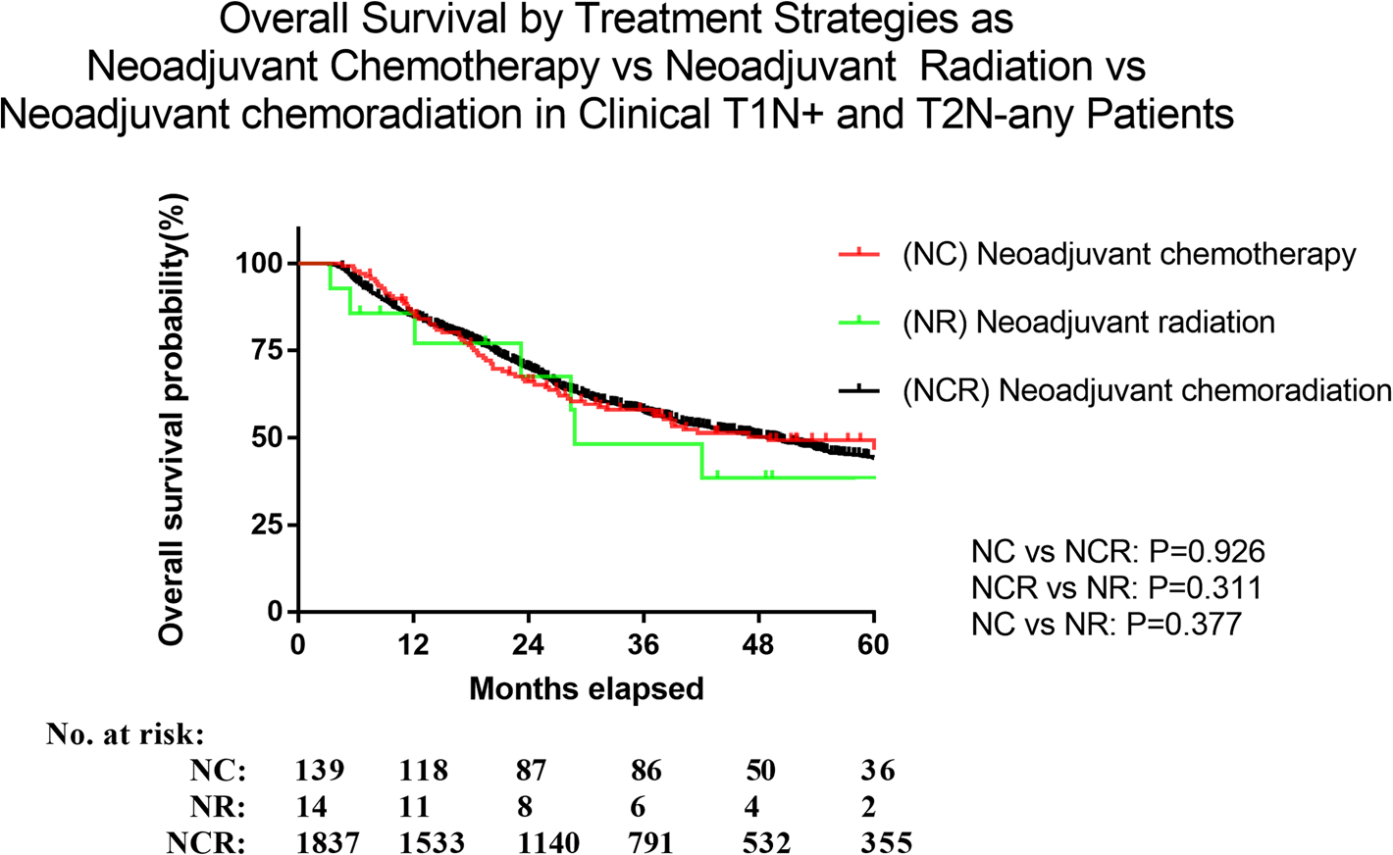
Neoadjuvant chemotherapy and neoadjuvant chemoradiotherapy cohorts were associated with a better trend for OS compared to neoadjuvant radiotherapy alone, but the difference was not significant (*P*=0.377, *P*= 0.311, respectively). There was no difference in OS between neoadjuvant chemoradiotherapy vs neoadjuvant chemotherapy alone (*P*=0.926)

**Fig. 4:**
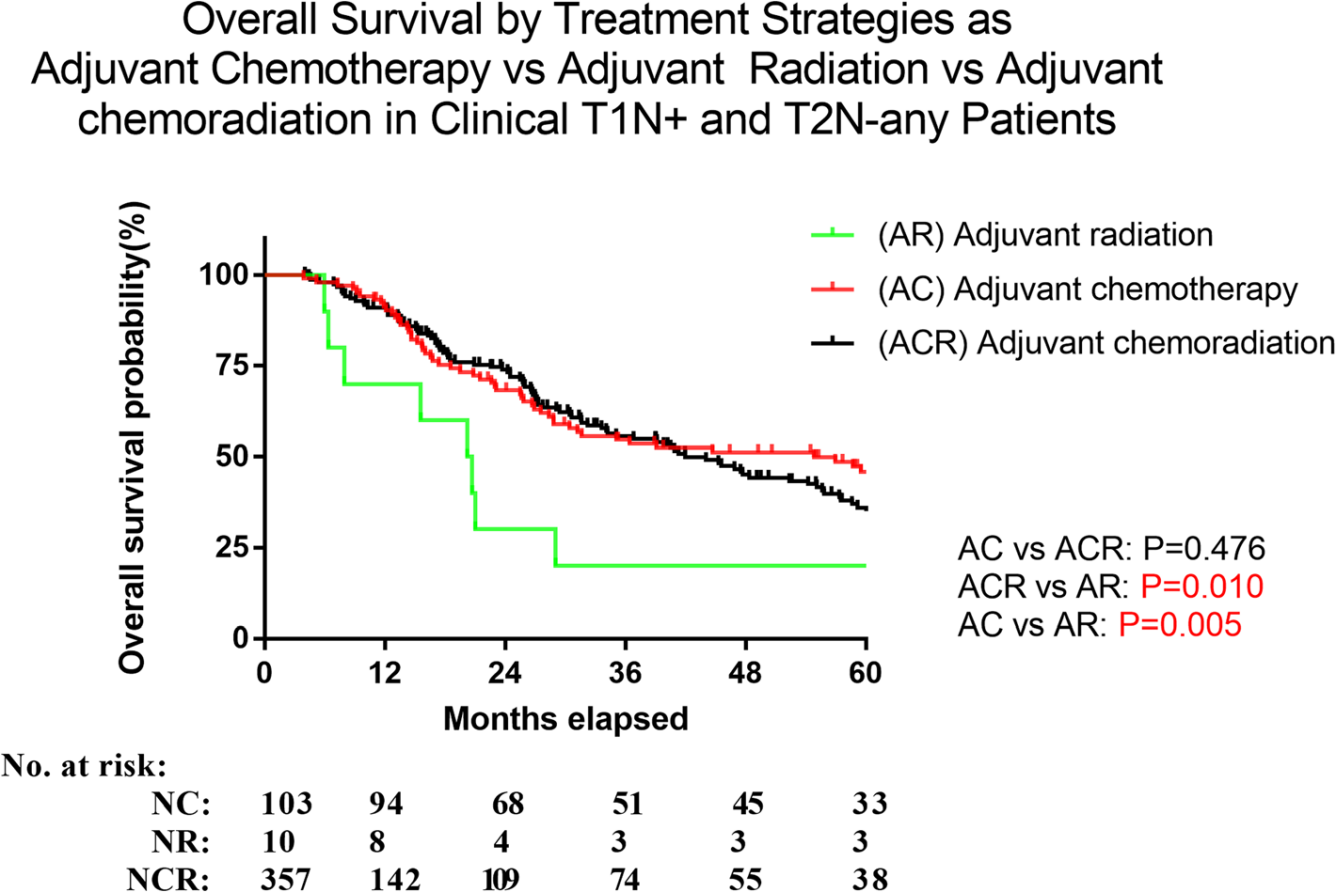
In patients only receiving adjuvant therapy after esophagectomy, there was no difference in overall survival between those who received adjuvant chemotherapy and adjuvant chemoradiation(*p*=0.476). Patients who received adjuvant radiotherapy alone after surgery was associated with a decreased overall survival when compared to both adjuvant chemotherapy(*p*=0.005) and adjuvant chemoradiation(*p*=0.01)

Further analysis was performed to find an optimal treatment strategy for patients who underwent NT and AT respectively. We initially compared the various neoadjuvant (NT) strategies. 92.8% had chemoradiation, 6.5% had chemotherapy and 0.7% had radiation in NT group; In this NT group, neoadjuvant chemotherapy and neoadjuvant chemoradiotherapy cohorts were associated with a better trend for OS compared to neoadjuvant radiotherapy alone, but the difference was not significant (*P*=0.377, *P*= 0.311, respectively). There was no difference in OS between neoadjuvant chemoradiotherapy vs neoadjuvant chemotherapy alone (*P*=0.926; Fig. 3). For patients in the adjuvant (AT) group, 58.6% had chemoradiation, 37.9% had chemotherapy and 3.5% had radiation in AT group. A survival benefit was associated in adjuvant chemotherapy and adjuvant chemoradiotherapy groups (*P*<0.005; *P*=0.010, respectively), compared to adjuvant radiotherapy alone. There were no significant differences noted in overall survival between adjuvant chemoradiotherapy and adjuvant chemotherapy alone (*P*=0.476; Fig. 4).

## Discussion

In this focused study, on clinical T1N+, T2 (both node negative and node positive) esophagus cancer patients, there was no evident superiority associated with neoadjuvant therapy over adjuvant therapy by multivariate analysis using Cox regression model. This relationship held true in both squamous cell carcinoma and adenocarcinoma groups. For T1N+ ESCC, this conclusion is limited by the relatively small number of patients in the NCDB cohort and may require additional studies.

The introduction of both chemotherapy and radiation in the neoadjuvant and adjuvant settings has positively impacted survival in patients with locally advanced esophageal cancer. There is significant literature on the use of multimodal therapy for the treatment of esophageal cancer and the strategies for treatment have been previously summarized [3, 5, 11]. There are proponents of various approaches and many reasons fuel this debate including difficulty in studying the disease due to anatomic and histologic heterogeneity. One major source of discussion is the use of neoadjuvant chemoradiotherapy versus neoadjuvant chemotherapy alone prior to surgery. Advocates for the use of chemoradiotherapy frequently quote the CROSS trial which showed significantly higher median overall survival in patients who received chemoradiotherapy followed by surgery when compared to surgery alone [10]. While these results of neoadjuvant chemoradiotherapy are quite remarkable, one of the criticisms of this study is the lack of a neoadjuvant chemotherapy arm in this study [14, 15]. The ability to achieve a R0 resection at the time of surgery is one of the most important factors for decisions on adjuvant therapies. In our study, we found a significant lower R0 rate associated with the AT only group when compared to the NT and CT groups. This is consistent with consensus that induction therapy will increase R0 rate at the time of surgery.

However, there is concern suggesting an increase in operative complexity and intraoperative and postoperative complications in the setting of neoadjuvant chemoradiation in an already complex operation of esophagectomy [15–17]. In a study by Reynolds and colleagues an increase in postoperative complications including respiratory complications were noted in patients with preoperative chemoradiation followed by esophagectomy, when compared with patients treated with surgery alone [17]. We also found significantly higher 30 and 90 day mortality in the NT group than that in the AT and the CT groups. The results support the idea that NT may have negative impact on surgical results. We need to present the harms and benefits of each of these options at the initial discussion or decision moment. We also need to be very cautious when interpreting above results. While the NCDB provides an extensive group of patients to study, it fails to capture patients who opted out of AT due to a protracted postoperative course. These patients have an associated higher 30d mortality and 90d mortality rate and their improper exclusion into the AT or CT groups may introduce some selection bias. Fortunately, there is only 5.13% of the patients, who should, but did not receive AT due to patient risk factors according to our analysis (ie, comorbid conditions, advanced age).

The sequence between chemotherapy and radiation with surgery has also been in debate. Accurate staging is important for selection of appropriate treatment strategy, however, there are significant inaccuracies in clinical staging modalities. One cohort where clinical staging with endoscopic ultrasound is notably inaccurate are patients with clinical T2 disease, where inaccuracies as high as 50% been reported [12, 13]. With the inaccuracies of clinical staging particularly for cT1N+ and cT2 tumors, upfront surgery, and subsequent treatment based on more reliable pathological staging information may be considered. In patients undergoing surgery first, their pathologic findings are definitive and provide definitive pathologic staging data. Therefore, this affords the multidisciplinary oncologic team to selectively choose the best treatment regimen based on final pathology. The role of adjuvant chemotherapy in esophageal cancer is not clearly established [5, 11]. However, a Phase2 study ECOG E8296 with adjuvant chemotherapy after completely resected adenocarcinoma showed encouraging results [5, 18]. Further, several meta-analyses investigated neoadjuvant vs adjuvant therapy in the setting of esophageal cancer with no significant differences in outcome [19, 20]. Speicher et al. compared induction therapy with patients that underwent surgery first in patients with clinical T2N0 disease [13]. While they were unable to show a benefit to receiving induction therapy in these patients, they were unable to further stratify the induction therapy as that data was not available until 2006 in the NCDB. In the current study, our findings also support the idea that there is a role for adjuvant therapy following surgery in various subgroups particularly those with cT1N+ and cT2Nany disease. Therefore, adjuvant therapy following surgery may be a viable treatment sequence for a select group of patients especially those with cT1N+ and cT2Nany disease. Ultimately, careful patient selection is necessary to identify those that would best benefit from this approach. This is highlighted by Semenkovich et al. who, through a decision analysis model, recommended the use of upfront induction in patients that are found to have high risk features for upstaging found on EUS [21].

There are limitations to note for the current study. First, this is a retrospective analysis utilizing a large administrative database, lacking of granular patient data, standardized staging or treatment regimens, and is therefore subjected to reporter and selection bias. In addition, the data being collected is limited and does not include important variables such as cancer-specific mortality data. Therefore, we were unable to evaluate any relationship between oncologic outcomes such as recurrence-free survival or have any insight into specific therapy regimens. Similarly, we were also unable to track specific intraoperative and postoperative complications with regards to surgery as well as the multi-modal therapies [22]. Additionally, Samson et al showed worse survival for upfront esophagectomy patients whom were upstaged with only 44.2% receiving adjuvant therapy and median overall survival of 27.5 months vs 43.9 months in neoadjuvant cT2 N0 patients. And so we acknowledged the concept of surgery first and allowing pathologic findings to determine adjuvant therapy has some risks [23]. Base on NCDB, we also found up to 60% patients who received chemoradiation therapy could not undergo esophagectomy. Nonetheless, the results of this study reflect outcomes based on the most up-to-date cohort of patients with T1N+, T2 (both node negative and node positive) esophageal cancer in US.

## Conclusions

In conclusion, in clinical T1N+, T2Nany patients, there was no evident superiority of NT over AT. We also found significantly higher 30 and 90 day mortality in the NT group than that in the AT and the CT groups. Surgery followed by adjuvant therapy can be considered to be an alternative option in these patients. Further prospective studies are needed to validate these findings.

## Supplementary Information

**Additional file 1: Supplemental Figure 1.** Flowchart of inclusion and exclusion criteria from National Cancer Database (2006-2015). In total, 2795 patients were identified and stratified into 3 groups according to the sequence of surgery (S) and additional therapy.

**Additional file 2: Supplemental 1.** Overall Survival by Treatment Strategies ( Neoadjuvant vs Adjuvant vs Combination Therapy) and different histology(ESCC vs EAC) in Clinical T1N+ and T2Nany Patients”. **Supplemental 2.** Multivariate analysis for overall survival in T2N0 patients using Cox regression model.

## Data Availability

All data generated or analyzed during the study are included in this published article.

## Abbreviations

NT: Neoadjuvant therapy
AT: Adjuvant therapy
CT: Combination therapy of neoadjuvant and adjuvant
ESCC: Esophageal squamous cell carcinoma
EAC: Esophageal adenocarcinoma
