# Supplementary figures and images for: The ideal approach for treatment of cT1N+ and cT2Nany esophageal cancer.: a NCDB analysis
Source: BMC Cancer. 2021 Dec 15;21:1334. doi: 10.1186/s12885-021-08896-0 (PMC8672500; doi:10.1186/s12885-021-08896-0)

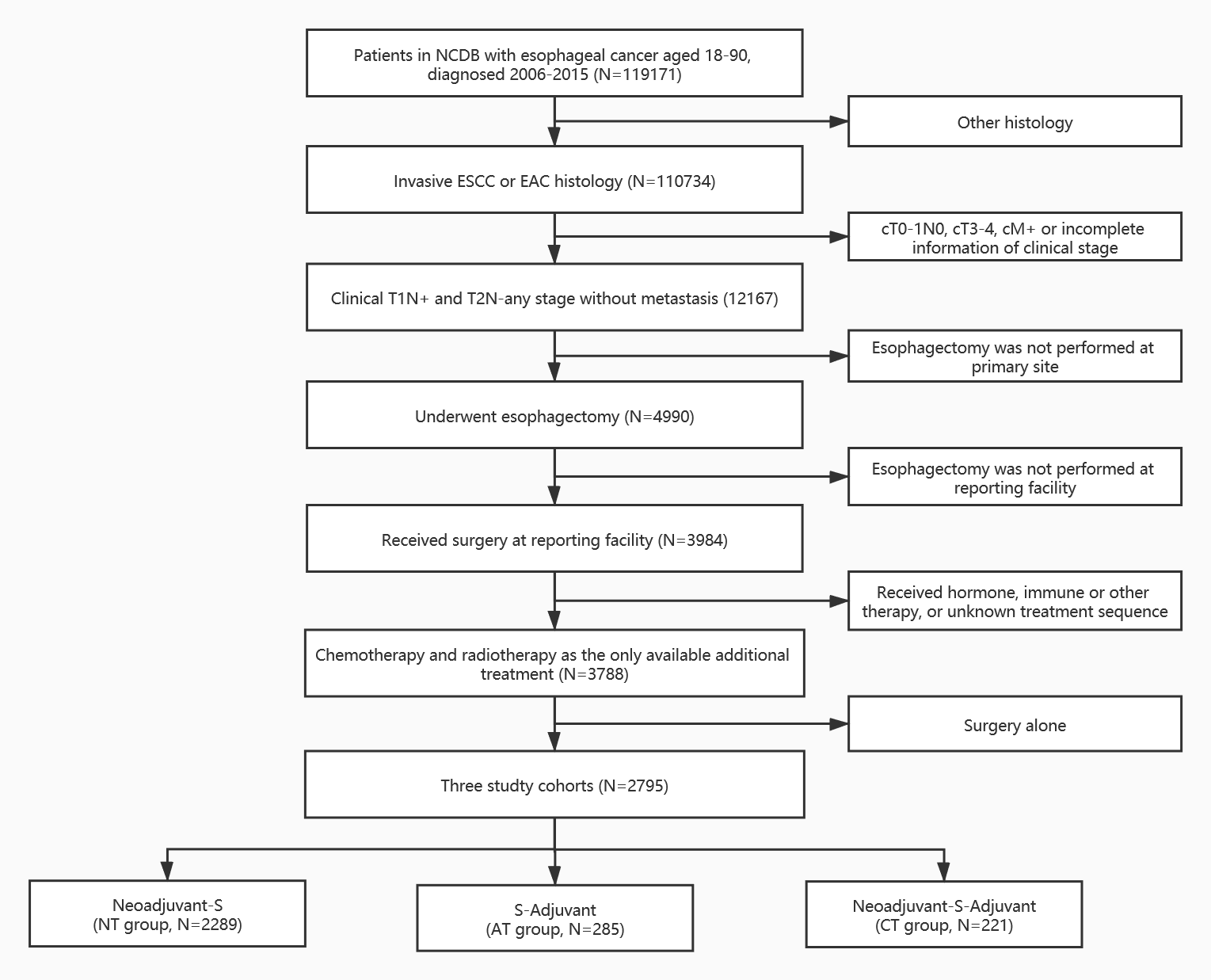

Supplement: Supplementary file 1 — Additional file 1: Supplemental Figure 1. Flowchart of inclusion and exclusion criteria from National Cancer Database (2006-2015). In total, 2795 patients were identified and stratified into 3 groups according to the sequence of surgery (S) and additional therapy. [file 12885_2021_8896_MOESM1_ESM.jpg]
