# Supplementary material for: The ideal approach for treatment of cT1N+ and cT2Nany esophageal cancer.: a NCDB analysis
Source: BMC Cancer. 2021 Dec 15;21:1334. doi: 10.1186/s12885-021-08896-0 (PMC8672500; doi:10.1186/s12885-021-08896-0)
Supplement: Supplementary file 2 — Additional file 2: Supplemental 1. Overall Survival by Treatment Strategies ( Neoadjuvant vs Adjuvant vs Combination Therapy) and different histology(ESCC vs EAC) in Clinical T1N+ and T2Nany Patients”. Supplemental 2. Multivariate analysis for overall survival in T2N0 patients using Cox regression model. [file 12885_2021_8896_MOESM2_ESM.docx]

| Supplemental 1 Overall Survival by Treatment Strategies ( Neoadjuvant vs Adjuvant vs Combination Therapy) and different histology(ESCC vs EAC) in Clinical T1N+ and T2Nany Patients”. | | | | | | | | |  |
| --- | --- | --- | --- | --- | --- | --- | --- | --- | --- |
|  |  | ESCC | | |  | EAC | | |  |
| Subgroups | Strategy | Adjusted HR | 95%CI  of HR | Adjusted P value |  | Adjusted HR | 95%CI of HR | Adjusted P value |  |
| Reference | NT | 1.000 | 1.000 | 1.000 |  | 1.000 | 1.000 | 1.000 |  |
| c Stage |  |  |  |  |  |  |  |  |  |
| T1N+ | AT | NAα | NAα | 0.279 |  | 0.622 | 0.273-1.415 | 0.257 |  |
|  | CT | NAα | NAα | 0.557 |  | 1.320 | 0.725-2.403 | 0.364 |  |
| T2N0 | AT | 1.350 | 0.700-2.605 | 0.371 |  | 1.079 | 0.823-1.416 | 0.580 |  |
|  | CT | 1.230 | 0.410-3.684 | 0.712 |  | 1.247 | 0.852-1.824 | 0.256 |  |
| T2N+ | AT | 1.697 | 0.729-3.952 | 0.220 |  | 1.041 | 0.716-1.515 | 0.833 |  |
|  | CT | 0.455 | 0.163-1.266 | 0.132 |  | 1.087 | 0.781-1.514 | 0.620 |  |
| α: Hazard ratio was not available for T1N+ ESCC in Cox regression model due to limitedvalid cases (2 NT and 2 CT). | | | | | | | |  |  |
|  | | | | | | | | | |

| **Supplemental 2 Multivariate analysis for overall survival in T2N0 patients using Cox regression model** | | | |
| --- | --- | --- | --- |
|  | **Adjusted HR** | **95%CI of HR** | **Adjusted P value** |
| **Age** |  |  | <0.001 |
| ≤55 | 1.000 (Refference) | 1.000 (Refference) |  |
| 56-65 | 1.005 | 0.802-1.259 |  |
| 66-75 | 1.303 | 1.015-1.673 |  |
| >75 | 2.181 | 1.649-2.885 |  |
| **Sex** |  |  | 0.050 |
| Male | 1.000 (Refference) | 1.000 (Refference) |  |
| Female | 0.808 | 0.652-1.000 |  |
| **Race** |  |  | 0.220 |
| White | 1.000 (Refference) | 1.000 (Refference) |  |
| Black | 1.062 | 0.743-1.519 |  |
| Others | 0.544 | 0.268-1.102 |  |
| **Insurance** |  |  | 0.102 |
| Private | 1.000 (Refference) | 1.000 (Refference) |  |
| Government | 0.794 | 0.402-1.568 |  |
| None | 0.971 | 0.489-1.929 |  |
| **Income 2008-2012** |  |  | 0.032 |
| <$48,000 | 1.000 (Refference) | 1.000 (Refference) |  |
| ≥$48,000 | 0.819 | 0.682-0.983 |  |
| **Education 2008-2012** |  |  | 0.238 |
| <87% | 1.000 (Refference) | 1.000 (Refference) |  |
| ≥87% | 0.899 | 0.754-1.073 |  |
| **UR 2013** |  |  | 0.105 |
| Metropolitan | 1.000 (Refference) | 1.000 (Refference) |  |
| Urban | 1.214 | 0.974-1.514 |  |
| Rural | 0.857 | 0.622-1.181 |  |
| **Charleson score** |  |  | 0.557 |
| 0 | 1.000 (Refference) | 1.000 (Refference) |  |
| 1 | 1.094 | 0.924-1.296 |  |
| ≥2 | 1.066 | 0.812-1.399 |  |
| **Year of diagnosis** |  |  | 0.182 |
| 2006-2010 | 1.000 (Refference) | 1.000 (Refference) |  |
| 2011-2015 | 0.899 | 0.768-1.051 |  |
| **Facility type** |  |  | 0.316 |
| Community | 1.000 (Refference) | 1.000 (Refference) |  |
| Comprehensive Community | 0.841 | 0.503-1.406 |  |
| Academic/Research | 0.752 | 0.454-1.248 |  |
| Integrated Network | 0.698 | 0.402-1.215 |  |
| Other or unknown |  |  |  |
| **Location** |  |  | 0.151 |
| Upper third | 1.000 (Refference) | 1.000 (Refference) |  |
| Middle third | 1.108 | 0.630-1.946 |  |
| Lower third | 0.853 | 0.485-1.500 |  |
| Overlapping | 0.687 | 0.333-1.418 |  |
| **Histology type** |  |  | 0.255 |
| ESCC | 1.000 (Refference) | 1.000 (Refference) |  |
| EAC | 0.869 | 0.682-1.107 |  |
| **Strategy** |  |  | 0.286 |
| Surgery alone | 1.000 (Refference) | 1.000 (Refference) |  |
| NT | 1.052 | 0.891-1.242 |  |
| AT | 1.185 | 0.926-1.516 |  |
| CT | 1.347 | 0.940-1.931 |  |
